# Trigonometric Regressive Spectral Analysis Reliably Maps Dynamic Changes in Baroreflex Sensitivity and Autonomic Tone: The Effect of Gender and Age

**DOI:** 10.1371/journal.pone.0012187

**Published:** 2010-08-16

**Authors:** Manja Reimann, Constanze Friedrich, Julia Gasch, Heinz Reichmann, Heinz Rüdiger, Tjalf Ziemssen

**Affiliations:** 1 Autonomic and Neuroendocrinological Laboratory, Department of Neurology, University Hospital Carl Gustav Carus, Dresden University of Technology, Dresden, Germany; 2 Research Group Neuro-Metabolism, Department of Neurology and Internal Medicine III, University Hospital Carl Gustav Carus, Dresden University of Technology, Dresden, Germany; Istituto Dermopatico dell'Immacolata, Italy

## Abstract

**Background:**

The assessment of baroreflex sensitivity (BRS) has emerged as prognostic tool in cardiology. Although available computer-assisted methods, measuring spontaneous fluctuations of heart rate and blood pressure in the time and frequency domain are easily applicable, they do not allow for quantification of BRS during cardiovascular adaption processes. This, however, seems an essential criterion for clinical application. We evaluated a novel algorithm based on trigonometric regression regarding its ability to map dynamic changes in BRS and autonomic tone during cardiovascular provocation in relation to gender and age.

**Methodology/Principal Findings:**

We continuously recorded systemic arterial pressure, electrocardiogram and respiration in 23 young subjects (25±2 years) and 22 middle-aged subjects (56±4 years) during cardiovascular autonomic testing (metronomic breathing, Valsalva manoeuvre, head-up tilt). Baroreflex- and spectral analysis was performed using the algorithm of trigonometric regressive spectral analysis. There was an age-related decline in spontaneous BRS and high frequency oscillations of RR intervals. Changes in autonomic tone evoked by cardiovascular provocation were observed as shifts in the ratio of low to high frequency oscillations of RR intervals and blood pressure. Respiration at 0.1 Hz elicited an increase in BRS while head-up tilt and Valsalva manoeuvre resulted in a downregulation of BRS. The extent of autonomic adaption was in general more pronounced in young individuals and declined stronger with age in women than in men.

**Conclusions/Significance:**

The trigonometric regressive spectral analysis reliably maps age- and gender-related differences in baroreflex- and autonomic function and is able to describe adaption processes of baroreceptor circuit during cardiovascular stimulation. Hence, this novel algorithm may be a useful screening tool to detect abnormalities in cardiovascular adaption processes even when resting values appear to be normal.

## Introduction

The arterial baroreflex is the primary mechanism involved in short-term cardiovascular control [1]. In reaction to variations in systemic arterial pressure arterial baroreceptors elicit opposing changes in efferent cardiovagal and sympathetic activities to adjust heart rate and peripheral vascular tone. The sensitivity of the baroreflex (BRS) arch is a dynamic parameter of sigmoid nature which is modulated by various intrinsic factors such as breathing, chemoreceptor reflexes, central stimuli and vessel wall elasticity [2]. Under conditions of sustained vessel wall distention, as during hypertension, BRS resets to lower values [3]. A pathological reduction of BRS has not only been associated with chronic blood pressure elevation [4] but also with other cardiovascular risk factors such as age [5], [6], [7], obesity [8], [9], arterial stiffness [10] and gender [6], [11]. An impairment of BRS has also been shown to predict outcome in cardiovascular diseases [12], [13]. Consequently, indexes of BRS have emerged as prognostic factor in cardiology [14], [15].

To date various methods for BRS determination are available which differ considerably in specificity, sensitivity and clinical applicability. In particular, invasive approaches like the modified Oxford method and the neck chamber method are often contraindicated under disease conditions. Non invasive methods encompass head-up tilting and Valsalva manoeuvre which are of low specificity since cardio-pulmonary receptors and vestibular circuits are likewise stimulated [16]. Therefore, modern computer-based methods measuring spontaneous fluctuations of RR intervals and blood pressure in the time or frequency domain are increasingly applied. Although the sequence method and spectral techniques based on Fast Fourier Transformation (FFT) are established methods of BRS determination they are hampered by the fact that sufficient frequency resolution can only be achieved using long data segments of more than 10 minutes [17], [18]. With longer duration of recording time the mandatory stationarity, describing a steady mean of the originally time-varying signals, becomes increasingly jeopardized. Furthermore, under disease conditions such as parkinsonism or arrhythmia long stable recordings are not always feasible or only fractional analyzable.

Accounting for these limitations we developed the algorithm of the trigonometric regressive spectral (TRS) analysis which uses statistic elements to cope with the stochastic nature of time-varying signals [19]. The data segments required for TRS analysis can be as short as 20 seconds allowing for dynamic evaluation of heart rate and blood pressure interaction over longer periods. Another advantage is that all oscillations are analyzed by the same (maximal) number of RR intervals thereby providing a high number of individual BRS values. This ensures a high confidence level of BRS determination which, along with short recording periods, may be of profound clinical relevance. The dynamic assessment of heart rate and blood pressure spectra by TRS allows a more precise evaluation of cardiovascular modulation under different settings [20], [21], [22], [23]. The aim of this study was to evaluate the ability of the TRS method to map age- and gender-related dynamic changes of autonomic function and BRS during exposure to cardiovascular stressors in a set of healthy individuals.

## Results

### Clinical characteristics of study participants

The body mass index was significantly lower in the young group compared to the middle-aged group (22.0±1.9 kg/m^2^ vs. 24.8±1.9 kg/m^2^, *p* = 0.001). Twelve participants were regularly smoking thereof five young and six middle-aged subjects. Two young and two middle-aged subjects were taking anti-allergic medication as needed and four middle-aged individuals were regularly using L-Thyroxin. Two middle-aged participants were on lipid lowering drugs (atorvastatin), one person was taking a proton pump inhibitor (esomeprazol) and another person was using pilocarpin eye drops.

### BRS and power spectral data in response to cardiovascular stimulation

#### Controlled breathing (Figure 1)

BRS and RRI significantly increased during controlled breathing at 0.1 Hz but remained unchanged upon controlled breathing at 0.2 Hz except in the group of middle-aged subjects. Controlled breathing did not evoke changes in systolic blood pressure. The total explaining power of RRI frequency spectra (RR-total weighed average variance) increased during breathing at 0.1 Hz but did not change upon breathing at 0.2 Hz. There was a shift from high towards low frequency oscillations during controlled breathing at 0.1 Hz while high frequency oscillations gained influence at a breathing pace of 0.2 Hz (data not shown). The prolongation of RRI during controlled breathing at 0.1 Hz was stronger in the middle-aged group while the decrease in high frequency power of RRI was more pronounced in the young subjects (age x time interaction).

**Figure 1:**
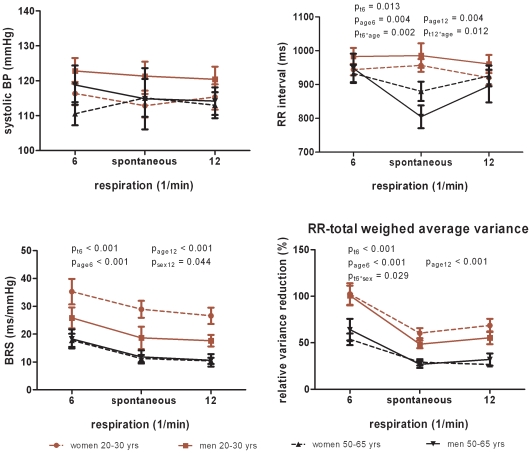
Baroreflex sensitivity, blood pressure and RR-total weighed average variance during controlled breathing. Data are mean and SEM; BRS, baroreflex sensitivity; BP, blood pressure; p_t_-values for repeated measures, p_age_ and p_sex_ from univariate analyses, and p for interaction effects (e.g. p_age*sex_).

#### Passive orthostasis (Figure 2)

Head-up tilting evoked significant reductions in BRS, RRI and systolic blood pressure. The changes in BRS and RRI were more pronounced in the young group (age x time interaction) and particularly in young women what BRS regards (age x time x sex interaction). High frequency power of RRI oscillations significantly decreased while there was a significant increase in low frequency power of RRI and systolic blood pressure oscillations as well as their frequency ratios. The young group exhibited a stronger shift from high to low frequency oscillations in heart rate (age x time interaction) during head-up tilting compared to the middle-aged subjects.

**Figure 2 pone-0012187-g002:**
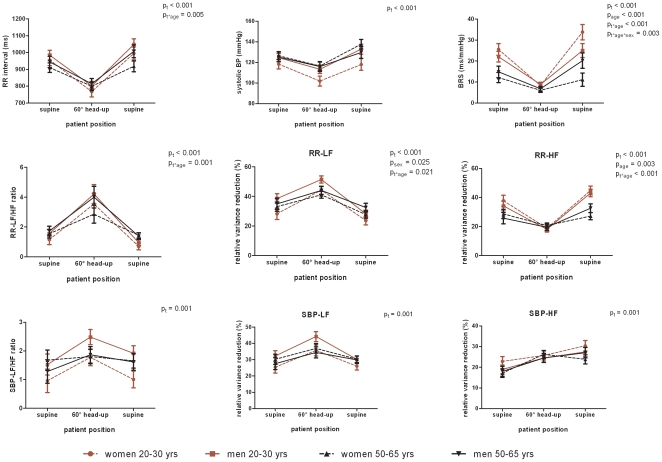
Baroreflex sensitivity and frequency spectra during head-up tilt. Data are mean and SEM; BRS, baroreflex sensitivity; Low frequency (LF) and high frequency (HF) power of RR interval and of systolic blood pressure (SBP); p_t_-values for repeated measures, p_age_ and p_sex_ from univariate analyses, and p for interaction effects (e.g. p_age*sex_).

#### Valsalva manoeuvre (Figure 3)

RRI and BRS decreased during the forced pressure phase while mean systolic blood pressure remained unchanged. Reductions of BRS were less pronounced with age (age x time interaction) particularly in middle-aged women (age x time x sex interaction). Forced pressure-evoked shortening of RRI was stronger in men than in women irrespective of age (sex x time interaction). There was a reduction in high frequency oscillations of RRI und systolic blood pressure. The low frequency component and the frequency ratio of both signals increased.

**Figure 3:**
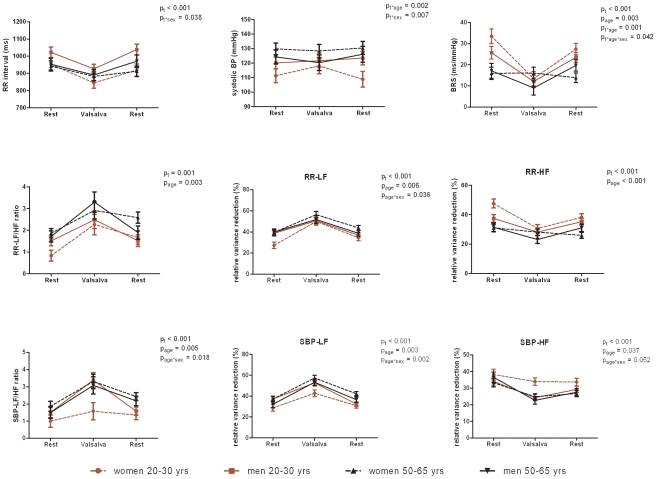
Baroreflex sensitivity and frequency spectra during Valsalva manoeuvre. Data are mean and SEM; BRS, baroreflex sensitivity; Low frequency (LF) and high frequency (HF) power of RR interval and of systolic blood pressure (SBP); p_t_-values for repeated measures, p_age_ and p_sex_ from univariate analyses, and p for interaction effects (e.g. p_age*sex_).

### Association between BRS and power spectral data (Figure 4)

**Figure 4:**
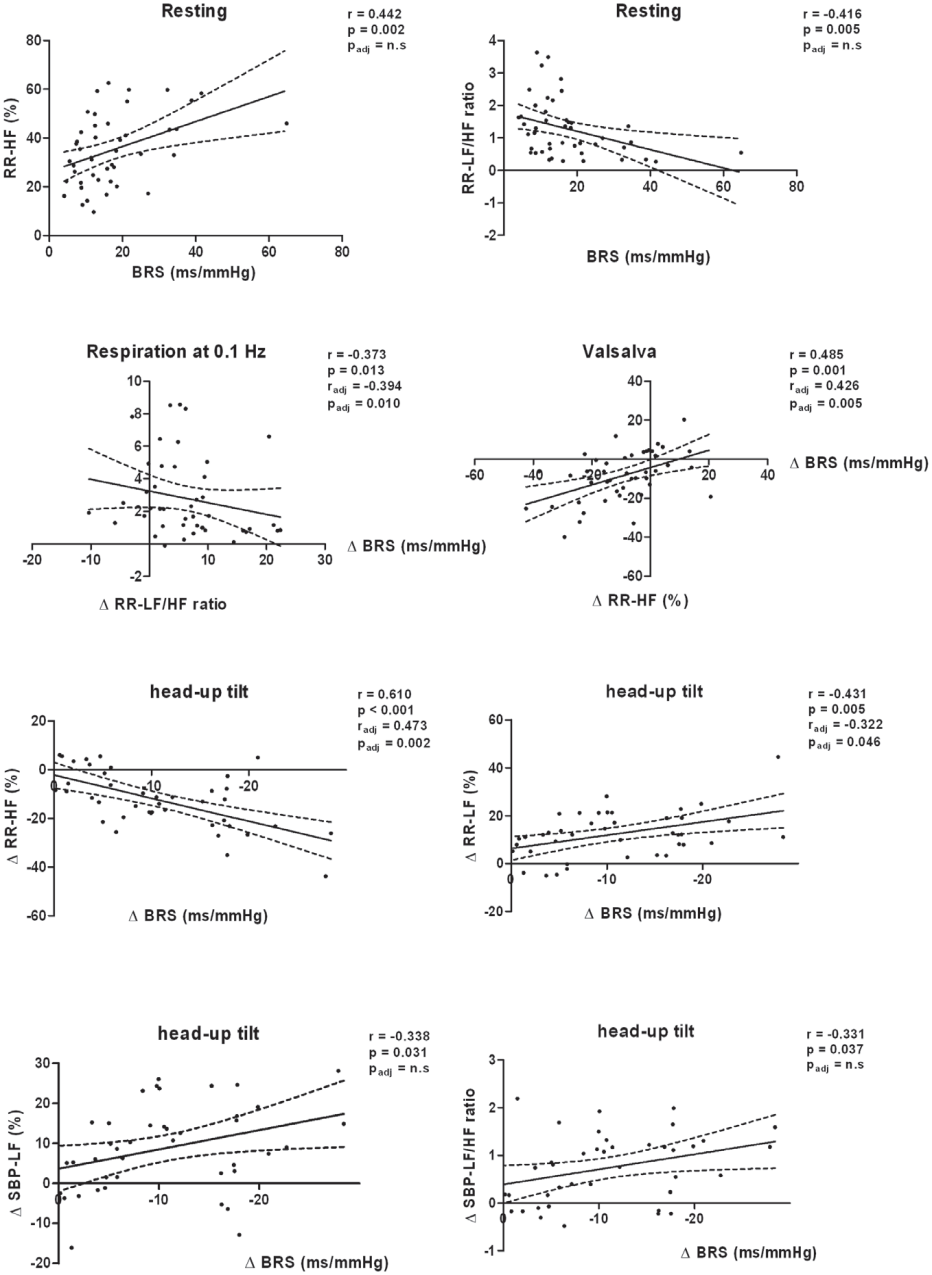
Associations of baroreflex sensitivity with frequency spectra. Data are unadjusted individual values; BRS, baroreflex sensitivity; Low frequency (LF) and high frequency (HF) power of RR interval and of systolic blood pressure (SBP); r, Pearson correlation coefficient; p value for unadjusted values; p_adj_ value after controlling for age and gender.

BRS was positively associated with high frequency power of RRI and negatively with the LF/HF frequency ratio of RRI. The association disappeared after controlling for age and gender. There was an inverse gender- and age-independent correlation between changes of BRS and the frequency ratio of RRI during controlled breathing at 0.1 Hz. Changes in BRS were positively correlated with changes in high frequency power of RRI during Valsalva manoeuvre. The association remained significant after adjusting for age and gender. Differences in BRS during head-up tilt were positively associated with changes in low frequency power spectra and frequency ratio of systolic blood pressure. An inverse correlation was found for changes in high frequency power of RRI. The associations of BRS with frequency power spectra of systolic blood pressure disappeared after controlling for age and gender.

### BRS and power spectral data by age

Spontaneous and stimulated BRS was significantly higher in the young subjects compared to the middle-aged individuals (Figures 1–3). In young individuals, RRI was significantly longer (lower heart rate) under resting conditions and controlled breathing (Figure 1). The total explaining power of RRI frequency spectra during rest and controlled breathing decreased with age. Likewise, the power of high frequency oscillations of resting heart rate reduced with age (Figures 1–3). There was an age dependency of low frequency fluctuations which was more pronounced in women than men (Figure 3). Frequency ratios of RRI and systolic blood pressure increased with age (Figure 3). The two latter features were not consistent across all measurements (Figure 2).

### BRS and power spectral data by gender

A marginal gender effect independent of age was observed for BRS upon controlled breathing at 0.2 Hz (Figure 1) and for low frequency power of RRI oscillations during orthostasis (Figure 2). Young women had significantly lower frequency power of resting systolic blood pressure oscillations compared to middle-aged women and men (Figure 1).

## Discussion

Owing to its predictive power for fatal cardiac events [12], [15] the measurement of BRS is increasingly introduced as risk stratification tool in clinical cardiology. Although a variety of methods for BRS determination are available, all of them are hampered either by their invasiveness, low specificity or by methodological limitations. We have previously described the novel TRS algorithm for non-invasive assessment of autonomic and baroreflex function based on spontaneous fluctuations of RRI and peripheral blood pressure [18], [19] where limitations of other BRS methods have been minimized. Since age and gender are the most important physiological correlates of BRS, explaining 52% of BRS variation [6], we focused our evaluation of TRS on age- and gender-related changes of BRS and frequency spectra in healthy individuals.

### BRS, autonomic tonus and age

In agreement with previous studies we observed a reduction of BRS with age [5], [6], [7]. This association was consistent across spontaneous and stimulated BRS values. A longitudinal investigation found a 3.6% annual attenuation of the spectrally determined intra-individual BRS [7]. Although not fully elucidated yet several mechanisms have been proposed underlying this age-related decline of BRS such as a reduced arterial compliance due to augmented collagen deposition, degeneration of elastin fibres, intima-media hyperplasia and progression of atherosclerotic disease [24], [25]. An age-related impairment of neuronal signal transduction pathways has also been suggested as a result of functional alterations in the central network, a reduced vagal activity or a diminished muscarinic receptor population of the sinus node [26]. Consistently, we and others [5], [27], [28] observed an age-dependent reduction in high frequency oscillations of resting heart rate, fluctuations that have been primarily ascribed to actions of the parasympathetic nervous system [29]. These high frequency fluctuations were positively and age-dependently associated with spontaneous BRS at resting conditions. Finally, BRS may also be modulated by an age-related decline in physical activity as well as increases in BMI [7] and blood pressure [24].

### BRS, autonomic tonus and gender

Previous studies reported a lower BRS and sympathetic tone in women compared to men [6], [11], [30], [31]. This observation, however, cannot be confirmed by our study. One possible explanation for the disparity may be the small sample size. This, however, seems rather unlikely since previous investigations evaluated groups of similar size [11]. More probably gender differences may have been masked by age-related differences of our mixed sample of younger and older subjects. This assumption is substantiated by the fact that the age-related decline in BRS was markedly in women but only moderate in the men. Consistent with our observation, Laitinen and colleagues (1998) reported that the gender difference observed in young subjects was lost with inclusion of older participants [6]. This gender by age interaction appears to underlie the modulation of BRS and autonomic function by sex hormones [32], [33]. Menopause-related shifts from cardiovagal to sympathetic predominance have been previously demonstrated [34]. In our study, a moderate to low impact of age in men is contrasted by strong effects in women. Thus, differences in autonomic tone between young men and women may converge with age explaining the lack of gender differences in our study.

### BRS, autonomic tonus and cardiovascular stimulation

Our findings match that of previous studies demonstrating an increased BRS during controlled breathing at 0.1 Hz [35], [36], [37], and a decreased BRS after postural change from lying to standing [23], [27], [38]. The improvement of BRS during deep slow breathing at 0.1 Hz has been, at least partially, ascribed to an increase in vagal tone and a concomitant decrease in sympathetic activity [35], [39]. Because we only observed slight increases in RRI and no changes in systolic blood pressure there must be still other mechanisms contributing to the increase in BRS. The paradox increase of heart rate fluctuations below 0.15 Hz presumably resembles a resonance phenomenon rather than a real change in autonomic tone. The origin of respiratory-related RRI fluctuations has long been a matter of debate with some arguing for baroreflex interaction while others proposing central mechanisms or breathing mediated stretch effects [35], [40]. Indeed, activation of the Hering-Breuer-Reflex has been associated with increases of BRS via reductions of the chemoreflex sensitivity [41]. Since we demonstrated an unchanged BRS upon breathing at a frequency of 0.2 Hz it seems that dynamic changes in BRS resemble a function of breathing frequency rather than regularization. To illustrate, during slow breathing the tidal volume rises concomitantly to ensure sufficient level of oxygen uptake. This deeper respiration resembles a stronger mechanical stimulus than breathing at a higher frequency thereby possibly modulating RRI fluctuations.

In contrast to deep breathing, the postural change from lying to standing and the Valsalva manoeuvre evoke much stronger changes in blood pressure which is reflected in an adaptive downregulation of BRS. Passive head-up tilting leads to pooling of blood in peripheral veins with concomitant reductions in central venous pressure, ventricular filling and stroke volume. As consequence, there is an activation of baroreflex circuit followed by sympathetically mediated peripheral vasoconstriction and cardiovagal withdrawal. Similarly, the sudden, transient increase in intrathoracic and intraabdominal pressure during Valsalva manoeuvre is followed by a drop in blood pressure which is counter-regulated by peripheral vasoconstriction and tachycardia. The baroreflex response to both manoeuvres was reliably mapped by TRS as demonstrated by an increase of low frequency power of systolic blood pressure and heart rate and a decrease of high frequency power of RRI. Previously, the decline in BRS during standing had been associated with the shift of autonomic tone towards sympathetic predominance [42]. In agreement, we found a negative association between changes of BRS and low frequency oscillations of blood pressure and RRI. It has been suggested that the increased sympathetic tone may lead to changes of mechanical vessel wall properties such as increased stiffness. Saeed et al. (2009)[38] demonstrated a smaller distention of the carotid vascular wall during standing after a comparable blood pressure change resembling a smaller stimulus for baroreceptor activation. We and others [6], [42] also revealed a positive association between changes in BRS and in parasympathetic activity (high frequency power of RRI) during postural change. Since parasympathetic activity is generally higher in supine position than in upright posture a higher BRS would be needed to compensate for blood pressure changes during lying. This relationship may possibly reflect a protection against hazardous blood pressure peaks during sleep. Still the regulation processes during passive orthostasis are very complex and may additionally involve changes in central signal transduction, in pulse pressure wave form, a reduction of carotid diameter and interaction between various pressure receptors systems.

Although drug intake was reportedly higher in the middle-aged group we consider the influence of an occasional intake of antihistamins, atorvastatin, esomeprazol and the local application of pilocarpin negligible. The intake of L-Thyroxin by four middle-aged individuals was deemed appropriate for normalisation of metabolic state. However, interferences of medication with autonomic function cannot be fully ruled out.

In summary, our findings convincingly demonstrate that the algorithm of trigonometric regressive spectral analysis is able to map age- and gender-related variations in baroreflex function and autonomic tone. More importantly, using TRS we were able to describe adaption processes of the baroreceptor circuit during cardiovascular stimulation. Therefore, TRS may be a useful screening tool to detect abnormalities in cardiovascular adaption processes even when resting values appear to be normal. Irrespective of the methodical advantages TRS may be ideally suited for use in clinical care because the evaluation of very short data segments from spontaneous fluctuations in heart rate and blood pressure render time-consuming, strenuous or invasive procedures for BRS determination unnecessary [20], [23].

## Materials and Methods

### Participants and Ethics

Fourty-five healthy volunteers were investigated in a cross-sectional study at the Department of Neurology of the University Hospital Dresden, Germany. The volunteers were recruited according to age. The young subjects (N = 23, 10 females and 13 males) had a mean age of 25±2 years and the middle-aged subjects (N = 22, 13 females and 9 males) were on average 56±4 years. Volunteers were excluded when they had a history of cardiovascular, neurological or metabolic disorders or when on regular medication affecting autonomic or cardiovascular function. All participants received detailed verbal and written information about the study objectives and procedure, and gave written informed consent. The study was approved by the Ethics Committee of the Faculty of Medicine of the Dresden University of Technology and the study procedure performed with the Declaration of Helsinki.

### Laboratory Autonomic Assessment

All recordings were performed in a temperature and humidity controlled specialized autonomic laboratory during the morning after a light breakfast. Consumption of caffeine or other xanthine containing foods were not permitted on the examination day. Alcohol and tobacco usage was ceased at the day preceding the measurements. Continuous cardiovascular monitoring was performed using the SUEMPATHY device (Suess Medizin-Technik, Aue, Germany) including the non-invasive blood pressure monitoring CBM3000 device (Nihon Colin Co, Komaki, Japan). The volunteers rested in a supine position on a tilt table for 20 min to reach cardiovascular steady state. At first, resting blood pressure and heart rate were recorded for 15 minutes. Thereafter, controlled breathing at 0.1 Hz (6/min) and 0.2 Hz (12/min) was performed for two minutes each. Following recovery, Valsalva manoeuvre commenced. To evoke an intra-thoracic pressure of 40 mmHg, the participant was instructed to take a large tidal breath and then to exhale for 15 seconds forcefully against a not completely closed valve connected to an aneroid pressure gauge [43]. Intra-thoracic pressure was recorded during the manoeuvre. The manoeuvre was repeated twice to obtain comparable recordings for analysis. For evaluation of orthostatic reaction, patients were tilted up to a 60 ° upright position within 15 seconds for five minutes during head-up tilt table testing.

### Trigonometric regressive spectral analysis

Cardiovascular data were analysed using TRS applied to corresponding values of systolic blood pressure and RR intervals (RRI) derived from systemic arterial pressure and ECG (sampling frequency of 512 Hz), respectively. The algorithm of the TRS provides a pure physiological spectrum based on trigonometric regression [18], [19]. All oscillations are detected under the condition that the deviance (y(t_i_) – Reg(t_i_))^2^ is minimal where y(t_i_) being the original RRI or systolic blood pressure and Reg(t_i_)  =  A x sin (ωt_i_ + ϕ_i_) being a trigonometric function of the parameter A (amplitude), ω (frequency) and ϕ (phase shift). Differently from FFT where frequencies ωt_i_ are of mathematical origin and depend on the length of the data segment T, TRS statistically fits each individual frequency optimally to a local data segment to achieve maximal variance reduction. Hence, TRS spectra match ideally the temporal RRI and blood pressure values irrespective of the length of the data segment. The optimal fit allows for the replacement of the relatively infrequent empiric individual BRS sequences of the sequence technique by a large number of statistically relevant TRS spectra.

Each predicted TRS oscillation reduces the total variance of the original process. This partial variance is subtracted from the total variance yielding the frequency related variance reduction expressed as percentage of the total variance. Upon reaching maximal variance reduction within a local data segment the amplitude and phase shift of the respective oscillation is calculated and then shifted beat by beat for temporal resolution of frequency and amplitude. Using a transfer function, the BRS is calculated from coherent oscillation pairs of RRI and systolic blood pressure in the respective frequency range (cross correlation coefficient >0.7). The EuroBavar Study demonstrated a good agreement of BRS calculated by TRS with that obtained by other techniques [21]. A summary of the algorithm can be found on the website of the European Working Group on Blood Pressure and Heart rate Variability (http://www.cbi.dongnocchi.it/esh-wg/).

### Baroreflex and Spectral analysis by TRS

Nonartifactual stationary and non-stationary global data segments of two minutes were analyzed using single time windows of 30 seconds. A global data segment of 15 seconds was analyzed during the forced pressure phase.

Spectral analysis allows for quantification of cardiovascular regulation by assessing spontaneous oscillations in systemic arterial pressure and RRI. Two main spectral bands are usually considered: High frequency oscillations of heart rate (RR-HF) (spectral band between 0.15 and 0.4 Hz) relate to respiratory sinus arrhythmia and, therefore, to parasympathetic cardiovagal tone [44]. High frequency oscillations of systolic blood pressure (SBP-HF) result from mechanical breathing-induced changes in stroke volume and are independent of autonomic activity. The other oscillation of interest is in the low-frequency range (spectral band between 0.04 and 0.15 Hz), usually centred around 0.1 Hz (6/min). Low-frequency oscillations of heart rate (RR-LF) are thought to reflect the baroreflex-mediated adjustments to the sinus node [45], [46] while low-frequency oscillations of systolic blood pressure (SBP-LF) are primarily the result of sympathetically mediated fluctuations in peripheral vasomotor tone. The LF/HF ratio allows for quantification of the relation between the two branches of the autonomic nervous system [46]. Spectral power describes the amount of variability of a signal or stochastic process at a specific frequency.

### Statistical methods

The SPSS software package version 16.0 for Windows (SPSS Inc., Chicago, IL, USA) was used for all statistical computations. Data are presented as mean ± SEM unless stated otherwise. Distribution of data was determined using Kolmogorov-Smirnov test. Logarithmic transformation was deemed appropriate when data did not meet the criteria of normality. Repeated measures ANOVA was applied to assess the effect of cardiovascular stimulation on BRS and spectral parameters. Parameters measured at different time points were treated as within-subjects variables. In case of more than one repetition the contrast for time was set <repeated>. The binary variables age group and gender were included as between-subjects factors to determine age and gender effects as well as interaction effects. Pearson correlation coefficients were calculated for absolute values and their changes. Differences were considered as significant when p<0.05.
